# The economic consequences of selected maternal and early childhood nutrition interventions in low- and middle-income countries: a review of the literature, 2000—2013

**DOI:** 10.1186/s12905-015-0189-y

**Published:** 2015-04-15

**Authors:** Nafisa Halim, Kathryn Spielman, Bruce Larson

**Affiliations:** Department of Global Health and Center for Global Health and Development, Boston University School of Public Health, 801 Massachusetts Ave, Crosstown Center, 3rd Floor, Boston, MA 02118 USA; Beth Israel Deaconess Medical Center, Boston, USA

**Keywords:** Reproductive, Maternal, Newborn, Child health (RMNCH) interventions

## Abstract

**Background:**

Globally, 25% of children aged 0 to 4 years and more than 10% of women aged 15 to 49 years suffer from malnutrition. A range of interventions, promising for improving maternal and child nutrition, may also improve physical and intellectual capacity, and, thereby, future productivity and earnings.

**Methods:**

We conducted a systematic literature review and summarized economic impacts of 23 reproductive, maternal, newborn and child health (RMNCH) interventions, published in 29 empirical studies between 2000 and 2013, using data from 13 low- and middle-income countries.

**Results:**

We find that, in low- and middle-income countries, RMNCH interventions were rarely evaluated using rigorous evaluation methods for economic consequences. Nonetheless, based on limited studies, maternal and childhood participation in nutrition interventions was shown to increase individuals’ income as adults by up to 46%, depending on the intervention, demography and country. This effect is sizeable considering that poverty reduction interventions, including microfinance and conditional cash transfer programs, have helped increase income by up to 18%, depending on the context. We also found, compared to females, males appeared to have higher economic returns from childhood participation in RMNCH interventions.

**Conclusions:**

Countries with pervasive malnutrition should prioritize investments in RMNCH interventions for their public health benefits. The existing literature is currently too limited, and restricted to a few selected countries, to warrant any major reforms in RMNCH policies based on expected future income impacts. Longitudinal and intergenerational databases remain needed for countries to be better positioned to evaluate maternal and early childhood nutrition intervention programs for future economic consequences.

## Background

Poor nutrition in childhood, including *in utero*, remains a public health challenge in many low- and middle-income countries. Globally, 25% of children aged 0 to 4 years and more than 10% of women aged 15 to 49 years suffer from malnutrition; and 90% of them live in 34 low-income countries [1]. In several low-income countries in Asia and sub-Saharan Africa, as many as 40% of women aged 15 to 49 years have short stature (less than 145 cm) or low body-mass index (less than 18.5 kg/m^2^). In low- and middle-income countries, child malnutrition accounts for 42% and 53% of all stunted and wasted children. Poor nutrition in childhood, including *in utero*, poses economic disadvantages for individuals, lasting for more than 30 years and transmitting across generations [2]. Fetal growth restriction and stunting in early childhood can reduce incomes in adulthood by up to 12% and 9%, respectively, depending on the context [2-5].

In recent years, several low- and middle-income countries have scaled up coverage of a wide range of reproductive, maternal, newborn and child health (RMNCH) interventions to ultimately prevent macro- and micronutrient deficiencies, short stature, or low body-mass index among women and children [1]. The RMNCH interventions in the areas of reproductive health and family planning, macro- and micronutrients supplementation, and disease prevention and treatment are believed to have the potential to improve not only nutrition but also earnings of service recipients. Underlying this presumption is a set of causal mechanisms: RMNCH interventions at the time of conception and during pregnancy may improve fetal-growth and birth weight in neonates [6,7]; optimal birth weight may lower risks of morbidity, mortality, impaired immune function, and poor cognition among neonates and infants, and stunting, wasting, later start of school, and lower school attainment and progression among children [6]; and, improved nutrition, cognition, and education in childhood may lead to improved nutrition and education in adolescents, and to improved labor capacity and productivity and earnings in adults [7,8].

Although plausible, conceptually, evidence from low- and middle-income countries still is emerging on to what extent RMNCH interventions can improve future productivity in education, economic and other activities. In this study, we conducted a review of the literature, and summarized the economic consequences of 23 reproductive, maternal, newborn and child health (RMNCH) interventions, published in 29 empirical studies between 2000 and 2013, using data from 13 low- and middle-income countries. To the extent RMNCH interventions are indeed economically beneficial, they will have policy implications for poverty reduction, especially, in 34 low-income countries, the home of 90% of malnourished women and children worldwide [9].

### Conceptual framework

**Figure 1:**
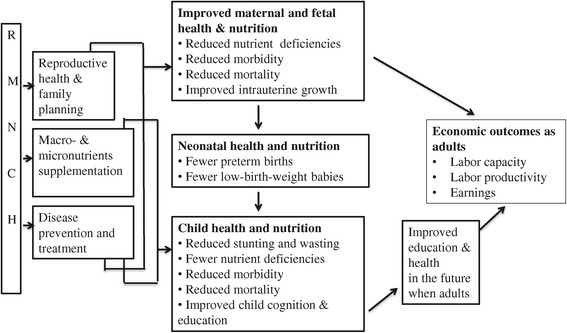
Possible pathways linking RMNCH interventions to future economic outcomes. Notes: RMNCH = Reproductive, maternal, newborn, child health interventions. BMI = Body Mass Index.

Figure 1 summarizes potential causal mechanisms linking RMNCH interventions, improved nutrition in childhood, including *in utero*, and future economic outcomes. For adults, higher earnings may follow from better human capital (i.e., cognitive skills, schooling, and stature) [7,8], where human capital accumulation may be shaped by adults’ nutritional status in childhood, including conditions *in utero*, weight and height at birth and during childhood years, and life-course decisions, including those related to fertility.

The RMNCH interventions can potentially raise human capital and earnings for women and for children, when they enter the labor market as adults. Access to reproductive and family planning services could raise women’s lifetime earnings by increasing her ability to control child births and, thereby, her opportunities to acquire skills and work in the formal sector [10]. Additionally, at the time of conception and during pregnancy, macro- and micronutrient supplementation (such as, food aid, iron supplementation) and disease prevention and treatment (such as, malarial treatment) may increase birth weight in neonates since they may improve nutrient and oxygen supplies to the fetus and prevent restricted fetal-growth and prematurity. Birth weight among neonates of greater than 1.5 kilograms may be associated with lower risks of mortality, morbidity, immune deficiencies, stunting and wasting among infants and children. By being on par in height- and weight for age, children may start school on time, progress through school on schedule, and have higher schooling attainment [6]. Improved nutrition, cognitive skills and schooling in childhood may lead to improved stature, cognitive skills and schooling in adolescents, and to improved work capacity, productivity and earnings in adults [7,8]. Additionally, conditions *in utero* may affect metabolism, which in turn increases risks of future obesity, health disease, and diabetes, and thereby, economic outcomes [11-13].

Additionally, across low- and middle-income counties, differential labor market conditions may determine to what extent RMNCH interventions can potentially contribute to future earnings. Despite having fewer earning opportunities, in general, low-income countries vary sizably among themselves in the extent to which their working-age male and female populations have access to earning opportunities. For example, during 2000–2012, in low-income countries, labor force participation ranged between 63-92% for working-aged males and 15-90% for females [14]. Therefore, for example, women may benefit more in terms of employment or income from participation in nutrition interventions in Bangladesh than in Afghanistan simply because female labor force participation is significantly different between the two countries (44 percentage points higher in Bangladesh compared to Afghanistan). Also, within a country, RMNCH interventions may differentially affect diverse demographic groups, depending on the country’s regional labor markets and within-country mobility, especially when children enter into the labor market as adults.

## Methods

### Databases for candidate studies

For selecting candidate studies for this review, we searched online bibliographic databases for peer-reviewed articles and working papers. For peer-reviewed articles, we searched in five online bibliographic databases: *EconLit*, *Social Sciences Citation Index (SSCI)*, *JSTOR*, *ScienceDirect,* and *PUBMED.* Also, we searched in three online bibliographic databases for working papers: National Bureau of Economic Research (NBER), Bureau for Research and Economic Analysis of Development (BREAD), and IDEAS. Finally, we searched bibliographies of identified articles and working papers. We conducted the search between April 10 and April 27, 2013.

### Search terms for identifying candidate studies

To produce a list of articles for the review, we searched for those RMNCH interventions, which potentially could raise earnings following the mechanism articulated in the Conceptual Framework in Section II. In particular, we searched for the RMNCH interventions in the areas of: reproductive health and family planning interventions; macronutrient supplementation; micronutrient supplementation; disease prevention and treatment. Also, for economic outcomes, we used search terms indicating indirect (i.e., human capital accumulation in the forms of cognition, education and health) as well as direct (i.e., income, wealth) economic outcome.

Therefore, we used combinations of the following search terms: (“nutrition interventions” OR “family planning” OR “folic acid fortification” OR “folic acid supplementation” OR “antenatal care” OR “essential package” OR “prevention and management of HIV and prevention of mother-to-child transmission in pregnancy” OR “prevention and management of childhood malaria” OR “early childhood development programs” OR “food aid” OR “integrated health and nutrition services” OR deworming OR “iron supplementation” OR “vitamin A supplementation” OR “zinc supplementation”) AND (“economic development” OR “economic growth” OR “economic consequences” OR “economic returns” OR welfare OR income OR wealth OR poverty OR socio-economic OR “human capital accumulation” OR “human capital formation” OR “human development” OR “human resources”) AND [(wom*n OR female*) OR (child* OR infant*)].

We searched using text words appearing in the title and/or the abstract of an article, along with using key words that have been indexed by the databases. We adjusted our search terms to include their synonyms, alternative spellings, and plurals. For example, we used wild cards (*) for alternative spellings and plurals of text words. Also, for possible synonyms of search terms, we used PubMed’s Medical Subject Heading (MeSH) thesaurus, which provides a listing of possible synonyms for each keyword indexed within it.

### Inclusion and exclusion criteria and study quality assessment

We included a study in the review if it met the following five criteria: (1) the study was a peer-reviewed article or a working paper; (2) the study was an empirical work; (3) the study included a measure of RMNCH intervention as a predictor variable; (4) the study included a measure of economic outcome (direct or indirect; see below for an explanation) as an outcome variable; (5) the study adjusted for confounders by constructing an appropriate comparison group (e.g., experimental study designs, or quasi-experimental study designs with appropriate statistical methods, such as instrumental-variable [IV], difference-in-difference [DID], and regression-discontinuity methods).

We excluded a study from review if it met any of the following criteria: the study (1) was a review, letter, or editorial work, (2) was a theoretical work, (3) was not published in English, (4) took place in an upper-middle or a high-income country for the year(s) considered in the study or (5) was published prior to the year 2000 or after 2013.

We applied the inclusion and exclusion criteria in two stages. First, during the online search stage, we selected the options in the online databases (e.g., publication date, publication type) that were commensurate with the criteria. Second, we read through the articles selected in the first stage, and further eliminated studies based on the exclusion criteria.

## Results

### Studies identified and selected

Our search for peer-reviewed articles and working papers in the included bibliographic databases provided an initial list of more than 200 studies. We screened titles and abstracts for relevance, and only 29 of the 200 studies remained as candidates for inclusion in this review. For these studies, we read again the abstracts as well as sections on research methods, and these 29 studies were included in the review. Of the four quality assessment criteria listed in section II.C., each article fulfilled criteria 1 and 2 and any one of criterion 3 or 4. Within these 29 articles, 23 distinct RMNCH interventions were evaluated. Across these 23 interventions, two were for reproductive health and family planning interventions, two for macronutrient supplementation, two for micronutrient supplementation, and seven were for disease prevention and treatment. Four interventions were targeted for the total population, which therefore included women of reproductive age and children. Across all 23 interventions, ten interventions were evaluated for direct economic impacts, while the other 13 interventions were evaluated for impacts on outcomes that are indirectly related to economic impacts.

### Populations of selected studies

The 29 studies in this review were carried out in thirteen different low- and middle-income countries across four economic regions: Bangladesh (5) and India (3) in South Asia; China (1), Thailand (1)and Indonesia (3) in East Asia and Pacific; Colombia (1), Costa Rica (1), Guatemala (4) and Mexico (1) in Latin America and Caribbean; and Botswana (1), Kenya (5), South Africa (1) and Tanzania (2) in Sub-Saharan Africa.

### Methodologies of selected studies

As required by inclusion criteria, all studies employed appropriate statistical methods for identifying program/intervention impact (typically average treatment effects).

Studies adopted a wide range of primary outcome measures. Twelve studies evaluated ten RMNCH interventions for direct economic impacts, including annual income, hours worked, hourly wages, and labor productivity. Seventeen studies evaluated the remaining 13 interventions for impacts on human capital accumulation in the form of: (a) schooling, such as enrollment, participation, performance tests, functioning, reading comprehension, and general knowledge; (b) cognitive development, such as, language and motor development, exploratory behavior, cognitive development and ability; and (c) health status.

### Findings

#### Reproductive health and family planning interventions

In low-income countries, 76% of pregnant women completed at least one visit for antenatal care, and 38% of married women aged 15–49 or their sexual partners use any form of contraception [14].

As summarized in Table 1, only three studies focused on reproductive health and family planning interventions were eligible for inclusion based on the selection criteria*.* Women who participated in family planning programs in Colombia as teenagers were 7% more likely to be employed in the formal sector as adults [15]. In Bangladesh, women participating in similar programs as teenagers earned one third more in wages per month as adults [16]. Moreover, these programs added 0.5 years of schooling among women aged 15–24 in Bangladesh and 15–19 in Colombia [16]. Also, in Bangladesh, family planning programs improved women’s weight by 0.79 kg, lowered lifetime fertility by 17%, improved birth spacing, and increased use of antenatal care by 0.395 [17].

**Table 1:** **Economic consequences of reproductive health and family planning interventions: key findings from studies in selected low- and middle-income countries, 2000—2013,** ***n*** **= 3**

**#**	**Study**	**Country**	**Study design**	**Sample**	**Statistical analysis**	**Economic impact: magnitudes and significance level**
1.	Miller, [15]	Colombia	Quasi-experimental	# of women 15—44: ~ 999,902	Probit	1. Women 15–19 employment **↑**: 7%*
						2. Women 20–24 employment **↑**: 4%*
2.	Schultz, [16]	Bangladesh	Quasi-experimental	# of villages: 141 # of HHs: 4,364	DID	1. Women 15—24 wages**↑**: 33%*
						2. HH assets **↑**: 25%*
						3. Men 15—24 wages: No impact
3.	Joshi and Schultz, [17]	Bangladesh	Quasi-experimental	# of villages: 141 # of HHs: 4,364 # of women 15—49: ~ 5,269	DID	1. Fertility ↓: 17%*
						2. Weight ↑:0.79 kg *
						3. Antenatal care use ↑: 40%*

*p ≤ 0.05. *DID* = Difference-in-Differences. **↑** indicates a positive impact; ↓ indicates a negative impact.

### Macronutrients supplementation

In low-income countries, poor nutrition affects 37% of children aged 0 to 4 years and more than 10% of women aged 15–49 [14]. For economic productivity and lifetime earnings, poor nutrition in childhood, including *in utero*, can be damaging: it reduces physical and intellectual capacity, and thereby, can reduce individual learning and economic productivity.

As summarized in Table 2, nutrition supplementation had the greatest economic returns in adulthood when children received nutritional supplementation while in *utero* (i.e., mothers received nutritional supplementation while they were pregnant with these children) and during the first three years of life. Hoddinott *et al.* [18] showed that, in Guatemala, a daily protein-enriched drink for children aged less than 3 years led to a 46% higher wage in their adult years. Furthermore, nutrition supplementation in early childhood was associated with a greater human capital accumulation in adults: in Guatemala, a daily protein-enriched drink for children aged less than 3 years was associated with an increased intellectual functioning in men [19] and women [19,20], and with reduced risks of chronic diseases in adults [21]. Also, in Kenya, a daily protein-enriched breakfast was associated with children’s higher attendance and better performance [22].

**Table 2:** **Economic consequences of macronutrients supplementation: key findings from studies in selected low- and middle-income countries, 2000—2013,** ***n*** **= 5**

**#**	**Study**	**Country**	**Study design**	**Sample**	**Statistical analysis**	**Economic impacts: magnitudes and significance level**
1.	Hoddinott *et al*., [18].	Guatemala	Experimental. A follow up study, using the INCAP *Oriente* Survey	# of villages: 4	OLS	1. Men’s wages ↑: 46% *
				# of individuals: 2,392		2. Women’s wages: No impact.
2.	Li *et al*., [20]	Guatemala	Experimental. A follow up study, using the INCAP *Oriente* Survey	# of women: 130	Ordinal Logit	1. Improved educational achievement (OR: 2.8*)
3.	Stein *et al*., [19].	Guatemala	Experimental. A follow up study, using the INCAP *Oriente* Survey	# of individuals: 1,448	GLM	1. Reading comprehension ↑: 3.46 points *
						2. Cognitive functioning ↑: 1.74 points*
4.	Stein *et al*., [21]	Guatemala	Experimental. A follow up study, using the INCAP *Oriente* Survey	# of individuals: 1,455	OLS; Logit	1. Men and women had a lower fasting glucose level (7.0 mg/dl*); systolic blood pressure (3.0 mm/dl*); triglyceride level (22.2 mg/dl*); and a higher density of lipoprotein cholesterol level (4.7 mg/dl*).
5.	Vermeersch and Kremer, [22]	Kenya	Experimental	# of schools: 50	Tobit, RE	1. School participation ↑: 30%*
				# of children: 2,392		2. Test scores ↑: 0.38 and 0.42*

*p ≤ 0.05. *OLS* = Ordinary Least Squares; *GLM* = Generalized Linear Models; *RE* = Random Effects. **↑** indicates a positive impact.

### Micronutrients supplementation

Globally, 42% pregnant women and 58% of children suffer from iron deficiency; and 32% of children suffer from iodine deficiency [14].

Indeed, as presented in Table 3, iron supplementation yields economic benefits to adults in Indonesia: a 6% income increase for women; and, for men, between 20 and 40% income increase; 0.8 more days worked per month; and 20 fewer loss of minutes per day in sleeping due to fatigue [23].

**Table 3:** **Economic consequences of micronutrients supplementation: key findings from studies in selected low- and middle-income countries, 2000—2013,** ***n*** **= 12**

**#**	**Study**	**Country**	**Population intervened**	**Study design**	**Sample size**	**Statistical analysis**	**Treatment**	**Economic impacts: magnitudes and significance level**
1	Thomas *et al.,* [23]	Indonesia	Adults, 30–70 yrs	Experimental	17,000	DID	Iron supplementation	1. Men:
								Income ↑: 20% *
								Hourly earnings↑: 40%*
								Productivity ↑: 0.8 days
								Minutes/day spent sleeping due to fatigue↓: 20
								2. Women: income↑: 6% *
2	Bobonis *et al*., [24]	India	Children, 2–6 yrs	Experimental	4,068	DID	Iron supplementation and deworming treatment	1. Weight ↑: 0.5 kg*
								2. School participation ↑: 5.8% percentage points*
								3. Effects most pronounced among girls and children of low SES.
3.	Stolzfus *et al*., [25]	Tanzania	Children, 6–59 months	Experimental	614	GLM	Iron supplementation and anthelmintic treatment	1. Language development ↑: 0.3—0.8 points *
								2. Motor skill development ↑: 0.4—1.1 point*
4.	Black *et al*., [26]	Bangladesh	Infants	Experimental	560	GLMM	Iron and zinc supplementation	Psychomotor Development Index score↑: 0.35*
5.	Field *et al*., [27]	Tanzania	Pregnant women	Quasi-experimental	1,395	FE	Iodized oil in utero	Schooling in years:
								1. Girls ↑: 0.82 **
								2. Boys ↑: 0.38 **
6.	O’Donnell *et al*., [28]	China	Pregnant women and children 2 yrs	Experimental	207	GLM; ANCOVA	Timing of initial iodine supplementation	Head circumference and Psychomotor Development Index scores:
								1. Children supplemented early in pregnancy those supplemented later*
7.	Schmidt *et al*., [29]	Indonesia	Pregnant women	Experimental	276	OLS	Micronutrient supplementation	No association with infants’ mental and psychomotor development.
8.	Tofail *et al*., [30]	Bangladesh	Pregnant women	Experimental	2,853	ANCOVA	Micronutrient supplementation; food supplementation	Infants:
								1. Problem-solving: ↑ 0.17*
								2. Psychomotor Development Index: ↑ 0.28*
								3. Behavioral ratings: ↑0.24*
9.	Prado *et al*., [31]	Indonesia	Pregnant women	Experimental	487	GLMM; GLM; RE	Micronutrients supplementation	Children
								1. Motor ability: ↑0.39*
								2. Visual attention: ↑0.24—0.37*
10.	Pongcharoen, *et al*., [32]	Thailand	Infants, 4–6 months	Experimental	560	GLMM	Iron and zinc supplementation	No impact on cognitive development
11.	Lozoff *et al*., [33]	Costa Rica	Infants, 12–23 months	Longitudinal survey of children who were treated with iron supplementation during infancy irrespective of their chronic iron deficient or good iron status.	191	ANCOVA	Iron supplementation	No impact on long-term behavioral and developmental outcomes
12.	Black *et al*., [34]	India	Infants	Experimental	221	ANOVA; GLM	Zinc and micronutrient-mix supplementation	No impact on cognitive and motor development

*p ≤ 0.05, **p ≤ 0.01. *ANCOVA* = Analysis of Covariance; *GLM* = Generalized Linear Models; *GLMM* = Generalized Linear Mixed Models; *FE* = Fixed Effects; *RE* = Random Effects. *DID* = Difference-in-Differences. SES = Socioeconomic status; **↑** indicates a positive impact; ↓ indicates a negative impact.

Furthermore, in childhood, iron supplementation led to greater human capital accumulation: preschool attendance increased in India [24]; motor skills improved in Tanzania [25] and in Bangladesh [26]. Maternal iodine supplementation led to higher cognitive abilities in children in Tanzania [27] and in China [28]: 0.6 years of more schooling in Tanzania and higher psychomotor test scores in China [27,28] the gains were larger for girls in Tanzania [27]. Maternal micronutrient supplementation led to improved cognition among children in Indonesia [31] and infants in Bangladesh [30].

However, maternal micronutrient supplementation did not improve cognition among another sample of infants in Indonesia [29]. Likewise, in childhood, iron, iron and zinc and zinc and micronutrient supplementation had no association was improved cognition in Thailand [32],Costa Rica [33], and India [34], respectively.

### Disease prevention and treatment

#### Malaria treatment

Globally, in 2012, 3.4 billion people were at risk of malaria, 207 million suffered and 627,000 died from it. As presented in Table 4, malaria eradication efforts led to a 0.8% and a 13.5% increase in income among adult men in India [4] and Mexico [5], respectively.

**Table 4:** **Economic consequences of malarial treatment: key findings from studies in selected low- and middle-income countries, 2000—2013,** ***n*** **= 2**

**#**	**Study**	**Country**	**Study design**	**Sample**	**Statistical analysis**	**Economic impacts: magnitudes and significance level**
1	Cutler *et al.*, [4]	India	Quasi-experimental using government map on malaria endemicity and the Indian National Sample Survey	# of men, 20—60: 111,218	DID	1. Men, HH expenditures ↑: 0.8%*
				# of women, 20—60: 107,551		2. Women, no impact on HH expenditures.
2	Venkataramani, [5]	Mexico	Quasi-experimental using state-level data on malaria death rates; the Mexican Family Life Survey	# of men, 20—60: 1,647	DID	1. Men, HH expenditures ↑: 12.2% *
				# of women, 20—60: 2,209		2. Women: no impact on HH expenditures.

*p ≤ 0.05. *DID* = Difference-in-Differences; **↑** indicates a positive impact**.**

#### Deworming

In Africa alone, up to 44 million women aged 14—49 years are infected with hookworms; this includes 7 million pregnant women. As presented in Table 5, deworming interventions can potentially increase individual labor productivity and earnings, along with reducing sick days and absenteeism. In Kenya, a deworming treatment of children with a single dose albendazole every six months or praziqunatel annually led to a 12% increase in work hours per week and to 0.1 additional meals’ consumption per day in their adult years [35]. The same intervention increased school attendance by six percentage points and schooling by 0.14 years (p), for boys of all ages and young girls aged less than 13 years benefitting more than older girls aged 13 years or older [36].

**Table 5:** **Economic consequences of deworming: key findings from studies in selected low- and middle-income countries, 2000—2013,** ***n*** **= 3**

**#**	**Study**	**Country**	**Study design**	**Sample**	**Statistical analysis**	**Economic impacts: magnitudes and significance levels**
1.	Baird *et al*., [35]	Kenya	Using the Kenyan Life Panel Survey, a follow up study of the Primary School Deworming Program, in which 75 schools (=32,565 pupils, aged 6—18 years) were randomly phased into the treatment of deworming medication	# of adults: 7,500	IV	1. # of hours worked ↑: 12%*
						2. Earnings ↑: 20%*.
2.	Miguel and Kremer, [36]	Kenya	Experimental	# of schools: 75 # of children 6—18: 32,565	OLS; IV	1. School participation ↑: up to 6.2 percentage points*.
3.	Gilgen *et al*., [37].	Bangladesh	Experimental	# of female adult workers: 553	OLS	No significant difference in labor productivity.

*p ≤ 0.05. *OLS* = Ordinary Least Squares; *IV* = Instrumental Variables; **↑** indicates a positive impact.

#### Aids treatment

Globally, in 2012, approximately 35.3 million people were living with HIV, 60% of them were women and children [38]; most of them live in sub-Saharan Africa. As presented in Table 6, in Kenya, ARV treatment led to a 20% and 21% increase in labor-market activities among HIV-infected men and women’s, respectively; HIV-infected men worked 35% more hours per week; and, once the HIV-infected adults within the household began treatment, young boys worked fewer hours in the labor market and increased their attendance at school [39]. Also, in Kenya, ARV treatment helped male tea pluckers offset decline in each of the total number of days per month worked in nonplucking assignments and the total number of days per month worked in plucking assignments by two days [40]. In Botswana, within a year of the initiation of ARV treatment, worker absenteeism decreased to 12 days from 20 days in the pretreatment year [41]. In South Africa, ARV treatment increased the likelihood of HIV-infected individuals actively seeking employment in the formal labor market [42].

**Table 6:** **Economic consequences of aids treatment: key findings from studies in selected low- and middle-income countries, 2000—2013,** ***n*** **= 4**

**#**	**Study**	**Country**	**Study design**	**Sample**	**Statistical analysis**	**Economic impacts: Magnitudes and significance levels**
**1**	**Thirumurthy et al.,** [39]	**Kenya**	**Quasi-experimental**	**# of individuals,18-65: 3,009**	**FE**	1. Labor force participation ↑: 20% *
						2. # of Hours worked ↑: 35% *
						**3. Young boys resumed back to school**
2.	Larson *et al.,* [40]	Kenya	Quasi-experimental	# of HIV-infect men tea plucker: 125 # of HIV-infect women tea plucker: 112	ITT	1. HIV-infected male and female tea-pluckers harvested 51% and 62% less tea, respectively, compared to healthy male and female tea-pluckers, respectively
						2. By the 24 months on ART, HIV-infected male tea-pluckers were 90% as productive as healthy male tea-pluckers; HIV-infected female workers were 80% as productive as healthy female tea-pluckers
3	Habyarimana, [41]	Botswana	Quasi-experimental	# of adults diamond mine workers: 441	OLS, FE	1. Absenteeism (=12 days) was comparable between HIV-infected on ART and healthy worker
						2. HIV-infected workers retained this rate of absenteeism for up to four years since ART initiation.
4	Coetzee, [42]	South Africa	Quasi-experimental	# of HIV-infected adults on ART = 237	AFTM; Cox Proportional Hazard Model	1. Time for transition from labor inactivity to actively looking for employment ↓ (*p* ≤ 0.05)

*p ≤ 0.05. *ITT* = Intent to Treat; *OLS* = Ordinary Least Squares; *FE* = Fixed Effects; *AFTM* = Accelerated Failure Time Models; **↑** indicates a positive impact; ↓ indicates a negative impact.

## Discussion

Starting early 1990s, Behrman and colleagues argued that, in low- and middle-income countries, policy makers should consider improving nutrition in order to improve wages and productivity among non-poor as well as the poor members of the society [43,44]. Echoing Behrman and his colleagues from 20 years earlier, Stenberg *et al*. [45] write in a *Lancet* article that, “improvement of preconception and maternal health, reduction of low birthweight and stunting through better nutrition, and expansion of a range of preventive child and adolescent health services are increasingly recognized as an investment in the potential for economic productivity and potential lifetime earnings in this and next generation”.

While the logic of these conclusions are well understood, the evidence from low- and middle-income countries remains limited on the extent to which such interventions might increase economic productivity and potential lifetime earnings. To support a better understanding of the evidence to date, and to consider needs for future research, we conducted a systematic literature review to summarize the economic impacts of reproductive, maternal, newborn and child health interventions in low- and middle-income countries. A total of 29 studies between 2000 and 2013 addressing 23 interventions met the inclusion criteria. We summarize key findings and limitations. Making policy tradeoffs between specific interventions is beyond the purpose of this review.

*First*, in low-income and lower-middle-income countries, nutrition interventions were *rarely* evaluated using appropriate evaluation methods for economic consequences, as evident by the small number of studies satisfying inclusion criteria. Between 2000 and 2013, six RMNCH interventions, including two for reproductive health and family planning, two for malaria, one for macronutrient supplementation, and one for deworming, were evaluated for economic consequences in six countries—Bangladesh, Colombia, Guatemala, Kenya, India and Mexico.

*Second*, when available, however, the limited number of studies finds that RMNCH interventions can increase individuals’ income as adults, depending on the outcome measure, intervention, demography and country. Women gaining access to reproductive health and family planning interventions increased women’s monthly wages by a third in Bangladesh [16] and labor force participation by 7% in Colombia [15]. An early childhood macronutrient intervention led to a 46% higher wage in adult years in Guatemala [18]. Deworming treatment for children led to a 12% increase in labor productivity in their adult years in Kenya [34]. Malaria eradication efforts for children led to a 0.8% increase in income in their adult years in India, and to a 13.5% increase in Mexico [4,5].

With an increase in earnings by between 7% and 46%, the economic returns of RMNCH interventions appear to be sizable, when they are compared, albeit crudely, with the economic returns of poverty reduction programs, namely, microfinance and conditional cash transfer programs. For example, poverty reduction programs were shown to have increased earnings by up to 18% for poor households in low and middle-income countries [46-52].

Third, compared to females, males appeared to have higher economic returns from participation in RMNCH interventions, perhaps because of a pro-male bias in the labor market. Malaria eradication led to increased income for adult men but not for women in India or in Mexico [4,5]. In Kenya, ARV therapy increased labor productivity more for men than it did for women [40]; ARV therapy reduced young boys’ weekly hours worked while increasing their attendance at school, but it showed no impact on young girls’ weekly hours worked and school attendance [39]. However, in Bangladesh, family planning intervention was associated with an approximate 33% increase in women’s wages, but it had no effect on men’s wages [16].

We recognize several limitations of this systematic review, many of which are also limitation of the existing literature. *First*, studies utilized a mix of direct and indirect indicators for economic outcomes, making comparisons of economic impacts across studies difficult. *Second,* because the studies were set in a variety of contexts (interventions and locations within countries), the ability to generalize findings is limited. *Third*, for 2000—2013 (the years of interest to this review), only three studies focused on reproductive health and family planning interventions were eligible for inclusion based on the selection criteria. This number is too small to provide persuasive evidence. *Fourth*, we have mitigated—but not eliminated—risks of a potential positive publication bias. We selected for review ten studies showing no effects on the study population [29, 32—34, 37] or a subsection [4,5,16,18] following our efforts to mitigate risks of a potential publication bias by expanding search databases to include working papers (see Section III.A). *Fifth*, we reviewed studies on AIDS treatment and not on AIDS treatment of pregnant women, which is a RMNCH intervention. Existing studies on PMTCT services did not meet our inclusion criteria. And *sixth*, except for Miguel and Kremer [36], studies were mute regarding externalities that might have caused under- or overestimates of impact. Miguel and Kremer [36] showed that, by attending schools within up to six kilometers away from schools receiving deworming treatment, students had up to 26% fewer infections.

## Conclusions

Investments today in reproductive, maternal, neonatal and child health may translate into future economic benefits among male and female populations in low- and middle-income countries. In high-income countries, a rich body of evidence has long documented these beneficial effects. For example, using a longitudinal dataset from the United Kingdom, Case *et al*. [53] find that prenatal and childhood health status are significant predictors of economic status in middle age (p. 368), even when parental- and individual-level confounders are adjusted for in analyses. Using data on monozygotic twins in the US and controlling for differences in genetics and family backgrounds, Behrman and Rosenzweig [54] find that lower-birth weight babies earn 6% less income in their lifetime than their higher birth weight counterparts.

In low- and middle-income countries, the existing literature currently is too limited, and restricted to a few selected countries, to warrant any major reforms in RMNCH policies based on expected future income impacts. Longitudinal and intergenerational databases on nutrition and economic indicators remain needed for countries to be better positioned to evaluate maternal and early childhood nutrition intervention programs for future socioeconomic consequences. Low and middle-income countries should prioritize investments in such longitudinal databases as the Institute of Nutrition of Central America and Panama longitudinal Study, the *Matlab* Health and Socioeconomic Survey, the Kenyan Life Panel Survey, and the Mexican Family Life Survey. Meanwhile, low- and middle-income countries should prioritize investments in RMNCH interventions for their public health benefits.
